# Immunological Enigma: A Case Report of COVID-19 Survival in a Patient With Human Immunodeficiency Virus, Hepatitis C Virus, and Tuberculosis Co-infection

**DOI:** 10.7759/cureus.69588

**Published:** 2024-09-17

**Authors:** Martina Bozhkova, Steliyan Petrov, Tanya Velyanova, Mariyana Stoycheva, Marianna Murdjeva

**Affiliations:** 1 Medical Microbiology and Immunology, Plovdiv Medical University, Plovdiv, BGR; 2 Infectious Disease, St. George University Hospital, Plovdiv, BGR; 3 Infectious Disease, Plovdiv Medical University, Plovdiv, BGR

**Keywords:** co-infection, covid-19, hcv, hiv, immunocompromised, survival, tb

## Abstract

This case report aims to elucidate the unique clinical course of a 34-year-old male patient diagnosed with human immunodeficiency virus (HIV), chronic hepatitis C, and prior tuberculosis (TB) infections, who subsequently contracted COVID-19. Immunological assessments revealed profound immunosuppression, marked by decreased CD4+ T cells (0.037 x 10⁹/L), alongside mildly elevated IgG levels (16.701 g/L), reflecting both HIV-related immunodeficiency and non-adherence to antiretroviral therapy (ART). Concurrently, the patient tested positive for SARS-CoV-2. Imaging findings demonstrated overlapping characteristics of TB and COVID-19. Timely initiation of specific TB therapy, alongside supportive care and optimized antiretroviral and anti-TB regimens, was implemented.

Despite the patient's immunocompromised state and complex medical history, he successfully recovered from COVID-19. Key factors contributing to survival included early TB diagnosis and treatment, comprehensive medical care, careful management of drug interactions, and a potentially effective individual immune response. Notably, no typical features of COVID-19 pneumonia were observed, suggesting that the dual infection may have influenced the clinical presentation. This case underscores the potential for positive outcomes in individuals with complex medical histories, including coexisting infections. Further research into the interplay of multiple infections in such patients is warranted to optimize clinical management strategies and enhance our understanding of COVID-19 within this distinctive population.

## Introduction

The COVID-19 pandemic, caused by the novel coronavirus, severe acute respiratory syndrome coronavirus 2 (SARS-CoV-2), has posed significant challenges to healthcare systems worldwide. While the majority of individuals infected with SARS-CoV-2 experience mild to moderate symptoms, certain populations, particularly those with underlying comorbidities or immunocompromised states, are at higher risk of severe illness and mortality [1-3]. Among these vulnerable groups, individuals with human immunodeficiency virus (HIV), hepatitis C virus (HCV), and tuberculosis (TB) infections represent a complex subset requiring special attention due to their compromised immune status [4,5].

TB is primarily transmitted via the airborne route, leading to pulmonary infection. The immune response to the infection is characterized by a complex interaction between innate and adaptive immunity. Following inhalation, *Mycobacterium tuberculosis* reaches the lungs, where it is generally engulfed by alveolar macrophages. However, the bacterium has evolved mechanisms to evade destruction by preventing the fusion of the phagosome with the lysosome, allowing it to persist intracellularly [6]. Macrophages detect a range of pathogen-associated molecular patterns (PAMPs) associated with *M. tuberculosis* through several cell surface and intracellular pattern-recognition receptors (PRRs), including Toll-like receptors (TLRs), C-type lectin receptors, NOD-like receptors, and the cyclic GMP-AMP synthase (cGAS)-STING pathway. These receptors initiate transcriptional responses in macrophages and modulate intracellular trafficking. TLR2 and C-type lectin receptors, upon detecting *M. tuberculosis*, activate NF-κB signaling, leading to the production of pro-inflammatory cytokines, such as tumor necrosis factor (TNF) and interleukin-6 (IL-6) [7].

In the regional lymph nodes, macrophages activate CD4+ T helper cells by presenting antigens in complex with MHC class II molecules. Th cells subsequently migrate to the site of infection, secreting cytokines like interferon-gamma (IFN-γ), which enhance macrophage activation by promoting phagosome-lysosome fusion and the production of reactive nitrogen and oxygen species. CD8+ T cells are also critical in controlling *M. tuberculosis*. Known for their cytotoxic activity, they recognize infected cells via MHC class I presentation by macrophages and dendritic cells. Upon activation, CD8+ T cells directly kill infected cells through the release of cytotoxic granules containing perforin and granzymes. Additionally, CD8+ T cells secrete IFN-γ, which enhances macrophage antimicrobial activity, and TNF, which facilitates granuloma development and infection containment.

Granulomas, the hallmark histological feature of TB, are composed of multinucleated giant cells, macrophages, epithelioid cells, and lymphocytes. Their formation serves to contain the infection and prevent dissemination. Nevertheless, *M. tuberculosis* can survive in a latent state within granulomas, with the potential to reactivate under immunosuppressive conditions [8].

HIV infection represents a significant cause of secondary immune deficiency, characterized by a complex immunopathogenesis that progressively damages the immune system, particularly CD4+ T cells. HIV disease progresses through three stages: acute HIV infection, clinical latency, and acquired immunodeficiency syndrome (AIDS), each marked by distinct immune features. HIV targets CD4+ T cells by binding to the CD4 receptor and co-receptors, CCR5 and CXCR4, and subsequently infects macrophages, which serve as reservoirs for the virus. The initial phase is typified by rapid viral replication, resulting in viremia, and is accompanied by a range of clinical manifestations, including fever, lymphadenopathy, rash, sore throat, and myalgia.

One of the earliest and most significant sites of CD4+ T-cell depletion is the gut-associated lymphoid tissue (GALT), which contains a large population of activated CD4+ T cells and is, therefore, highly susceptible to HIV infection. Plasmacytoid dendritic cells (pDCs) play a pivotal role in the immune response during this phase, functioning as antigen-presenting cells. They migrate to lymph nodes via the expression of the CCR7 homing receptor, which directs them to these immune hubs. Upon reaching the lymph nodes, pDCs activate CD4+ T cells by presenting viral antigens, thereby contributing to the initial immune response against the virus [9]. During the acute phase, the immune system responds primarily with cytotoxic CD8+ T cells (CTLs), which are responsible for controlling viral replication. Additionally, pDCs contribute to the shaping of the cytokine profile through the production of type I interferons, creating a pro-inflammatory environment that further activates immune cells [10].

In this phase, HIV-specific antibodies are typically absent or present in low quantities, resulting in what is known as the "window period," during which an HIV antibody test may return negative despite high levels of viremia. As the infection progresses to the clinical latency stage, the immune system re-establishes partial control of the virus, resulting in a reduction in viral load. During this phase, ongoing low-level viral replication causes chronic immune activation, exhausting CD8+ T cells and gradually reducing CD4+ T-cell counts, particularly in peripheral blood. HIV-specific antibodies eventually become detectable during seroconversion, although they are insufficient for the complete elimination of the virus. pDCs continue to influence immune responses during this phase, modulating T-cell activation and cytokine production. However, their functions may become impaired over time due to chronic HIV-induced immune dysregulation.

AIDS, the final stage of HIV infection, is defined by a CD4+ T cell count below 200 cells/μL or the occurrence of opportunistic infections [11]. Those in this stage are susceptible to infections and neoplastic diseases, including Kaposi's sarcoma, non-Hodgkin's lymphoma, as well as conditions such as pneumocystis pneumonia, TB, and cytomegalovirus infection.

The HCV has been identified as a significant factor in the development of liver cirrhosis and hepatocellular carcinoma. In cases of chronic HCV infection, the actions of host immune factors and viral proteins contribute to the persistence of the virus and the dysregulation of the immune system. Cytotoxic T cells and natural killer (NK) cells are responsible for the destruction of HCV-infected hepatocytes through the action of perforin and granzyme B, while the non-cytolytic clearance process is driven by IFN-γ. The interaction between the HCV and the host immune system is a determining factor in whether the infection resolves or becomes chronic. During HCV infection, viral RNA activates receptors such as Toll-like receptors (TLR3, TLR7), RIG-I, and MDA5, thereby triggering the production of type I and III interferons [12]. These interferons inhibit the replication of the HCV and activate NK cells, which produce IFN-γ and tumor necrosis factor-alpha (TNF-α) to mature dendritic cells (DCs) and further suppress viral replication. Dendritic cells, in turn, release IL-12, which prompts CD4 and CD8 T cells to become Th1 and cytotoxic T cells, respectively [13]. Th1 cells produce IL-2, IFN-γ, and TNF-α, which promote CD8 T cell proliferation and inhibit HCV replication without cell destruction. Additionally, IFN-γ facilitates the maturation of B cells into plasma cells, which subsequently produce neutralizing antibodies.

In recent years, the co-occurrence of multiple infectious diseases in a single patient has become an emerging concern, demanding a comprehensive understanding of the interplay between these pathogens and the host's immune response. This case report presents a remarkable observation of a patient diagnosed with HIV, HCV, and TB who was subsequently admitted to an infectious disease clinic with an additional diagnosis of COVID-19. Contrary to expectations, the patient successfully recovered from the COVID-19 infection despite the presence of three comorbidities that could potentially exacerbate the severity of the disease.

To date, limited data exist regarding the clinical outcomes and immune responses in patients with multiple comorbidities, including HIV, HCV, TB, and COVID-19. The aim of this case report is to present a unique clinical scenario, where an individual with this triad of infections survives a COVID-19 infection despite their immunocompromised state. Understanding the mechanisms underlying this unexpected outcome may shed light on potential protective factors, guide clinical management strategies, and contribute to the growing body of knowledge on COVID-19 in immunocompromised individuals.

In this report, we describe the clinical presentation, laboratory findings, and therapeutic interventions implemented for this patient. Furthermore, we discuss potential immunological mechanisms and host factors that might have contributed to their successful recovery. We hope that this case study will encourage further research and prompt healthcare providers to consider individualized approaches when managing COVID-19 in patients with multiple comorbidities, emphasizing the importance of a comprehensive understanding of the immune response in this unique scenario.

## Case presentation

A 34-year-old male patient was diagnosed with HIV 16 years prior, in October 2007. At the time of diagnosis, his virological parameters indicated an HIV load of 7,220 viral copies/mL and a CD4+ T-cell count of 501 cells/μL. In 2019, he initiated antiretroviral therapy (ART) with emtricitabine/tenofovir disoproxil, 1 tablet (containing 200 mg of emtricitabine and 300 mg of tenofovir disoproxil fumarate) once daily (a nucleoside reverse transcriptase inhibitor), and raltegravir, 800 mg twice daily (an integrase inhibitor that helps prevent viral DNA from integrating into the host genome), with poor adherence to the treatment. The patient also had co-existing chronic hepatitis C, likely due to previous intravenous drug use.

In 2017, the patient was diagnosed with TB based on a positive sputum smear for acid-fast bacilli and positive inoculation. TB treatment included rifampin, isoniazid, ethambutol, and pyrazinamide. On April 25, 2021, the patient presented to the infectious diseases department with symptoms of astheno-adynamia, weight loss, cough, chest pain, and a fever of 38°C. The patient appeared cachectic and in a moderately severe general condition, with severe non-albicans oropharyngeal candidiasis. Vital signs showed tachycardia with an average heart rate of 110 beats/min, blood pressure of 90/60 mmHg, and oxygen saturation of 99% on room air (SpO2). A chest examination revealed the presence of rhonchi and wheezes, but no lymphadenopathy was observed. Nasopharyngeal swab analysis was performed using real-time PCR (RT-PCR), which confirmed a SARS-CoV-2 infection.

**Figure 1:**
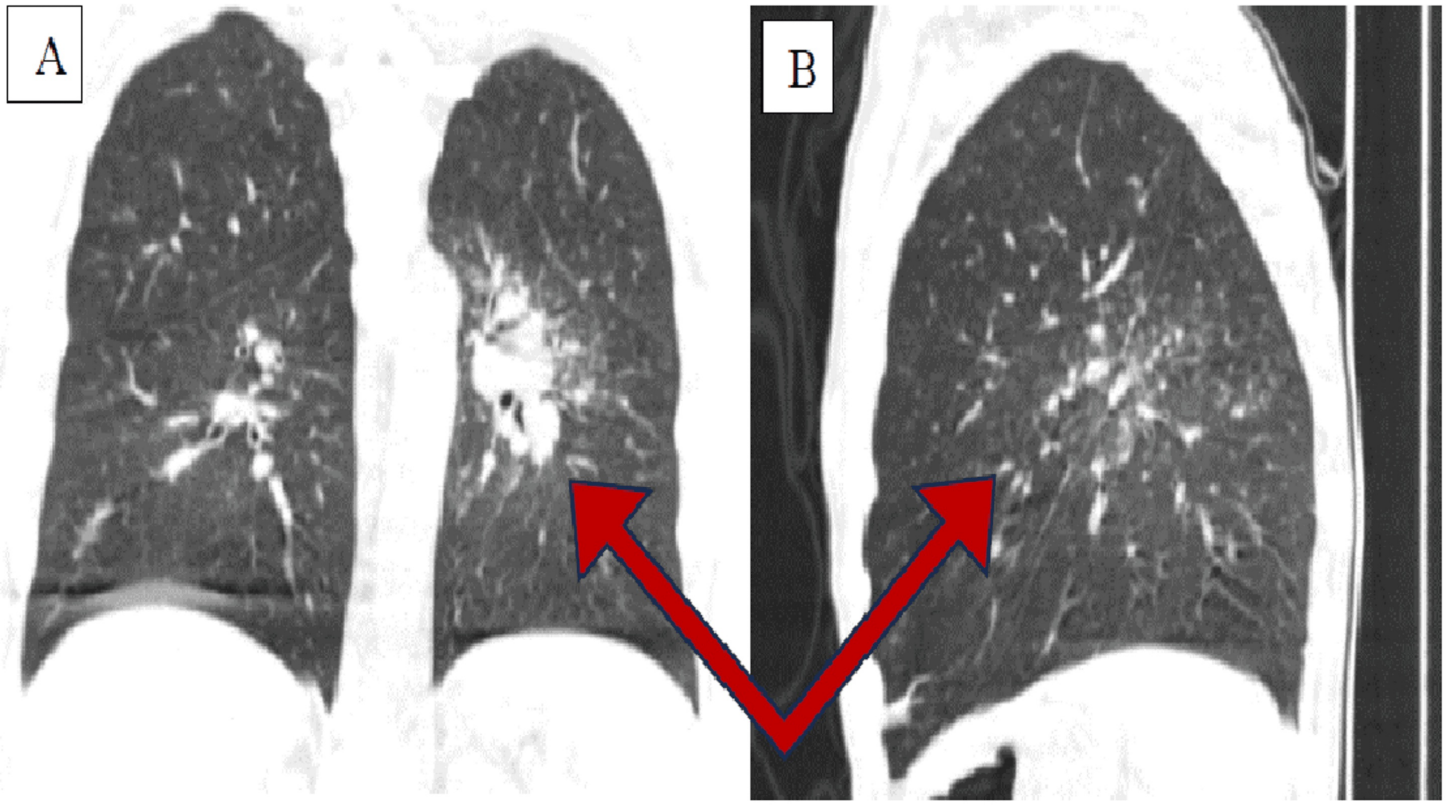
Computed tomography (CT) scan of patient’s lungs showing disseminated nodular focci: (A) front view; (B) side view

Upon admission to St. George University Hospital in Plovdiv, the patient exhibited moderate anemic syndrome, neutrophilia with lymphopenia (ratio: 3.23), an erythrocyte sedimentation rate of 90 mm/h, mildly elevated liver enzymes, hypoalbuminemia, dyselectrolytemia, and increased levels of D-dimers and fibrinogen (Tables 1, 2). A CT scan revealed disseminated nodular foci (Figure 1A, B), primarily in the 6th segment and to a lesser extent in the first and second segments of the left thoracic half. Interstitial changes were observed in the same segments, along with bundles of lymph nodes in the mediastinum (para-aortic, sub-aortic, and subcarinal) and the left hilus. No typical changes such as subpleural sparing, ground-glass opacification/opacity, or interlobular septal thickening, indicative of COVID-19 pneumonia, were detected.

**Table 1:** Results from patient’s complete blood count, indicating prominent anemia, leukocytosis, and lymphopenia

Laboratory parameters	Patient’s results	Reference ranges
Hemoglobin (HGB)	117 ↓	140-180 g/L
Erythrocytes (RBC)	4.28 ↓	4.5-6 10^12^/L
Hematocrit (HCT)	0.34 ↓	0.40-0.54 L/L
Leukocytes (WBC)	10.5 ↑	3.5-10.5 10^9^/L
Platelets (PLT)	198	140-400 10^9^/L
Erythrocyte sedimentation rate (ESR)	63 ↑	2-15 mm/h
Neutrophiles	90.2 ↑	42-70%
Eosinophils	0.2	0-6%
Basophils	0.2	0-2%
Monocytes	4.0 ↓	5.8-11.8%
Lymphocytes	5.4 ↓	22-48%

**Table 2:** Results from patient’s biochemical analysis, indicating dyselectrolytemia, hypoalbuminemia, and elevated liver enzymes levels

Laboratory parameters	Patient’s results	Reference ranges
Potassium (К)	4.1	3.5-5.6 mmol/L
Sodium (Na)	129 ↓	136-151 mmol/L
Clorine (Cl)	92 ↓	96-110 mmol/L
Urea	4.9	3.2-8.2 mmol/L
Creatinine	86	74-134 µmol/L
Total protein	62	60-83 g/L
Albumin (ALB)	23 ↓	35-52 g/L
Glucose (GLUC)	5.4	2.8 – 6.1 mmol/L
Total bilirubin (TBIL)	15.5	3.4 - 21 µmol/L
Aspartate aminotransferase (ASAT)	69 ↑	0-50 U/L
Alanine transaminase (ALAT)	58 ↑	0-50 U/L

Microbiological and immunological tests confirmed infection with *M. tuberculosis *through direct microscopy of sputum (Ziehl-Neelsen staining for acid-fast bacilli) and positive sputum culture. Additionally, a positive IFN-γ-based T-Spot.TB test was conducted to evaluate the specific immune response against *M. tuberculosis*.

Immunological parameters revealed increased levels of immunoglobulin G (IgG) at 16.701 g/L (reference range: 6.1-16.1 g/L), while IgA at 4.154 g/L (0.8-4.9 g/L) and IgM at 0.985 g/L (0.3-2.4 g/L) were within the reference intervals. Total immunoglobulin levels were measured using turbidimetry. Cytokine levels (Interleukin-10, Interleukin-33, Interleukin-28A, CD40L) were evaluated using enzyme-linked immunosorbent assay (ELISA), indicating increased levels of IL-10 and IL-33, while IL-28A and CD40L were within normal values (Table 3). Cytometric bead array was performed to evaluate the Th1/Th2/Th17 cytokine profile. Results from the assay showed increased levels of IL-6 and IL-10, while other biomarkers (IFN-γ, tumor necrosis factor-α, IL-2, IL-4, IL-17a) were within reference ranges (Table 4). Tests for perinuclear anti-neutrophil cytoplasmic antibodies (pANCA), anti-neutrophil cytoplasmic antibodies (cANCA), and anti-endothelial cell antibodies (AECA) were negative. Flow cytometry (Table 5) showed a significant decrease in the absolute count of CD4+ T cells (5 cells/μL) and CD19+ B cells (3 cells/μL), with a slight increase in CD8+ T cells (115 cells/μL).

**Table 3:** Patient’s coagulation status

Laboratory parameters	Patient’s results	Reference ranges
Activated partial thromboplastin time (APTT)	22.1 ↓	24-35 seconds
D-dimer	1.73	0-0.50
Thrombin time (ТТ)	17.4	14-21 seconds
Fibrinogen	6.38 ↑	2.0-4.5 g/L
Prothrombin time %	71.4	70-120%
Prothrombin time, INR	1.1	0.7-1.1
Prothrombin time	12.4	10-14 seconds

**Table 4:** Patient’ s cytokine profile

Immunological parameters	Patient’s results
Interleukin-10 (pg/mL)	1.71
Interleukin-33 (ng/mL)	6.07
Interleukin-28A (ng/mL)	132.5
CD40L (ng/mL)	1.32
Interleukin-2 (pg/mL)	1.31
Interleukin-4 (pg/mL)	1.54
Interleukin-6 (pg/mL)	9.7
Interleukin-17a (pg/mL)	0
Tumor necrosis factor (pg/mL)	0
Interferon-γ (pg/mL)	1.11

**Table 5:** Patient’s flow cytometry results, depicting pronounced depletion of CD4+ T-lymphocytes and CD19+ B-lymphocytes

Immunological parameters	Patient’s results	Reference ranges
Lymphocytes (× 10^9^/L)	1.121	1.0–2.8
CD3^+ ^T-lymphocytes (× 10^9^/L)	0.928	0.7–2.5
CD4^+^ T-helper cells (× 10^9^/L)	0.037 ↓	0.4–1.6
CD8^+ ^T-cytotoxic cells (× 10^9^/L)	0.852	0.2–1.1
CD19^+^ B-lymphocytes (× 10^9^/L)	0.014 ↓	0.1–0.7
CD16^+^CD56^+^ NK cells (× 10^9^/L)	0.180	0.1–0.7
CD3^+ ^T-lymphocytes (%)	83	59–85
CD4^+^ T-helper cells (%)	3 ↓	28–60
CD8^+ ^T-cytotoxic cells (%)	76 ↑	11–38
CD19^+^ B-lymphocytes (%)	1.2 ↓	6–13
CD16^+^CD56^+^ NK cells (%)	16	4–26
CD4/CD8 ratio	0.04 ↓	0.9–3.6

The patient's therapy included trimethoprim/sulfamethoxazole 800 mg/160 mg twice a day, fluconazole 100 mg once a day, low-molecular-weight heparin at a prophylactic dose, hepatoprotective medications: silymarin, ademetionine, and vitamin B6. On May 1, 2021, specific TB therapy was initiated, consisting of rifampicin 600 mg once a day, ethambutol 1000 mg once a day, pyrazinamide 1500 mg once a day, isoniazid 300 mg once a day, and levofloxacin 500 mg once a day. On May 18, 2021, ART was restarted. The patient was discharged with an improvement in overall well-being, no febrility, no signs of respiratory failure during hospitalization, and a negative anti-SARS-CoV-2 PCR test.

## Discussion

In this case report, we present a challenging clinical scenario of a 34-year-old male patient with a complex medical history, including HIV infection, chronic hepatitis C, and previous TB infection, who was diagnosed with COVID-19 in the setting of poor adherence to ART.

The patient's immunological status was characterized by a significant decrease in CD4+ T cells and CD19+ B cells, alongside slightly increased levels of IgG, presenting a typical picture of immunosuppression due to both HIV infection and non-adherence to ART. This immunosuppressive state likely contributed to the development of severe oropharyngeal candidiasis and susceptibility to opportunistic infections, such as TB. Furthermore, while CD4+ cells are the primary target of HIV, there is evidence that B cells are also indirectly affected by the virus, leading to their exhaustion, which may explain the low numbers observed in our patient. Another phenomenon caused by HIV is the polyclonal expansion of B cells, which could contribute to elevated serum IgG levels [14].

The detection of SARS-CoV-2 in the patient highlights the possibility of concurrent COVID-19 infection in individuals with underlying immunocompromised conditions. Several studies have established that immunocompromised individuals, including those with HIV and chronic hepatitis C, are at increased risk of severe COVID-19 and adverse outcomes [15,16].

The co-existence of active TB further complicated the patient's clinical course. TB is a leading cause of morbidity and mortality among HIV-infected individuals, and the combination of HIV and TB can lead to a reciprocal interaction, accelerating the progression of both diseases [17].

The imaging findings of disseminated nodular foci and interstitial changes in the lungs, along with bundles of lymph nodes in the mediastinum, were consistent with known manifestations of both TB and COVID-19 [18]. However, it is important to note that no typical changes indicative of COVID-19 pneumonia were detected in this case, suggesting that the co-infection might have influenced the clinical presentation of COVID-19.

Comparing our findings with other similar case reports in the literature, there have been documented instances of COVID-19 infection in immunocompromised individuals with underlying HIV infection [19] and TB [18]. Some case reports have also discussed the management challenges of co-infections in patients with chronic viral infections, such as hepatitis C and COVID-19 [15]. These reports emphasize the importance of optimizing antiretroviral and anti-TB therapy in patients with dual infections to improve outcomes. Some authors also discuss the possibility of a concurrent TB infection serving as a protector against severe COVID-19 through innate immunity mechanisms [20]. Another emerging hypothesis is the protective role of the Bacillus Calmette-Guérin (BCG) vaccine in COVID-19 [21].

The survival of the patient despite having HIV, chronic hepatitis C, TB, and COVID-19 raises several interesting points for discussion. The key factors contributing to his survival could be the following:

Timely diagnosis and treatment

Early detection and prompt initiation of specific TB therapy likely played a crucial role in controlling the TB infection and preventing further complications.

Medical care and treatment

The patient received supportive care, including antifungal and hepatoprotective medications, which may have helped manage the non-albicans oropharyngeal candidiasis and chronic hepatitis C. The patient's medical team would have played a crucial role in his survival. Specialized care for individuals with complex medical histories, including close monitoring, appropriate antiviral treatments, oxygen therapy, and other supportive measures, can significantly improve outcomes in severe COVID-19 cases.

Individual immune response

It is essential to recognize that each patient's immune response can vary significantly, even in the presence of similar comorbidities. Despite his health challenges, it is possible that the patient's immune system mounted a strong and effective response against the SARS-CoV-2 virus. Although HIV and chronic hepatitis C can weaken the immune system, not everyone with these conditions experiences a complete loss of immune function. Some individuals may retain partially functional immune responses that allow them to control certain infections to some extent. It is also possible that, due to the compromised immune system, the patient did not mount an overactive response typical of severe COVID-19, which could have served as a protective mechanism, sparing the body from further damage.

Drug-drug interactions

Close monitoring and management of potential drug-drug interactions among the different therapies administered were likely important to prevent adverse reactions and treatment complications. Due to the hepatotoxic, neurotoxic, and nephrotoxic side effects of some of the medications, hepatoprotectors and vitamin B6 were administered.

Serendipity and chance

Sometimes, patients with seemingly insurmountable odds can overcome severe illnesses due to a combination of unpredictable factors, often attributed to serendipity and chance.

## Conclusions

It is essential to remember that survival in such complex cases is a combination of multiple factors working together. The immune system's resilience, access to appropriate medical care, timing of interventions, and individual genetic differences can all play significant roles in determining outcomes. One important consideration is the need to look at the bigger picture when treating a patient, rather than focusing solely on the most obvious clinical manifestations. There is always the possibility of co-morbidities, especially in patients with HIV. Each case is unique, and drawing generalized conclusions from individual cases can be challenging. This highlights the importance of ongoing medical research in the fields of immunogenetics and pathogen-host interactions to better understand the interplay between various medical conditions and their impact on COVID-19 outcomes.
